# Comparative Analysis of the Surface Roughness of Class V Composite Restorations Using a Conventional Polishing System and Pre-contoured Cervical Matrices: An In Vitro Study

**DOI:** 10.7759/cureus.45901

**Published:** 2023-09-25

**Authors:** Neha N, Hima Sandeep A, Sarita Bhandari, Pradeep Solete, Sahil Choudhari

**Affiliations:** 1 Conservative Dentistry and Endodontics, Saveetha Dental College and Hospitals, Saveetha Institute of Medical and Technical Sciences, Saveetha University, Chennai, IND

**Keywords:** polishing, roughness, pre-contoured, matrix, composite

## Abstract

Background: Rough surfaces of restorations have an impact on the accumulation of plaque, unaesthetic contour, and marginal adaptation, thereby affecting their functional, aesthetic, and clinical performance and the long-term stability of the restoration. Several polishing systems are currently in use for composite restorations, but information on their impact on surface roughness is limited. The present study aimed to determine the surface roughness of class V composite restorations polished using pre-contoured cervical matrices and to compare it with a conventional disc polishing system.

Materials and methods: Twenty maxillary anterior teeth were collected and used in the study. Class V cavity preparation was done, and the cavities were restored with two types of composites (microhybrid and nanohybrid) from commercially available brands (Ivoclar and Dentsply) and finished and polished using two polishing systems (super-snap and pre-contoured cervical matrices). The evaluation of surface roughness was done using an atomic force microscope (AFM).

Results: The surface roughness parameters (Ra-arithmetic mean/average line roughness and Sa-average surface roughness) of the class V cavities restored using pre-contoured cervical matrices were significantly lower for both the tested resin composites.

Conclusion: The surface roughness of Class V cavities restored using pre-contoured cervical matrix systems was significantly less for both microhybrid and nanohybrid composites.

## Introduction

Composite resins have significantly advanced the field of conservative and aesthetic dentistry. They consist of a polymer matrix that is organic in nature, filler particles that are inorganic and a coupling agent. Variations in these constituents can influence their polishing effectiveness, consequently resulting in variations in the surface roughness among different composite materials [1]. Finishing and polishing composite resins are important steps in restorative dentistry. The aesthetic and functional clinical performance of composites depends not only on their structure and shade but also on the adhesive system used and the finishing and polishing protocol employed [2,3]. Unpolished surfaces tend to have a rough and irregular surface, which can be sensed by the tip of the tongue, leading to patient discomfort [4]. They also have a higher affinity for plaque accumulation, affecting gingival health and harmony and causing gingival irritation, leading to marginal gingivitis and eventual progression into periodontal disease [5]. Improper contour and poor marginal integrity will result in eventual staining, leading to poor esthetics, lesser wear resistance, and failure of the restoration [6]. Microbial adhesion to the rough surfaces of restorations is the primary etiology of secondary caries [7].

Surface roughness is one of the important parameters that affects the clinical performance and longevity of the composite restoration [8]. Resin composites are highly demanded by patients due to aesthetic concerns and are hence widely employed [9]. Composite resin, if finished and polished in the correct sequence, can have better longevity and clinical performance than a natural tooth and can be aesthetically pleasing to the patient as well [10]. Pre-contoured matrices, tin foil matrices, window matrices, cellophane strips, etc. have attained popularity in recent times. Cervical matrices are transparent pre-contoured matrix forms that are commercially available in various forms for anterior and posterior tooth restorations. They are employed for light-curable resins, glass ionomers, and resin-modified glass ionomer cement [11]. They fit the exact contour of the tooth and compress the material while curing or hardening, giving the resulting restoration a good contour and marginal adaptation. It is a properly shaped material that forms the missing wall of the tooth structure, against which the restorative material can be injected or condensed for restoration to achieve a natural shape and dimension [12].

Various finishing and polishing systems for class V restorations are available, including carbide burs, abrasive diamond points and disks, aluminum discs, rubber cups, mylar strips, polyester strips, abrasive-impregnated polishing pastes, etc. [13]. During polishing with any abrasive system, the filler particles may be exposed, loosened, or fractured, leading to surface roughness. Surface roughness can be depicted through various parameters that are in use, but the most common parameters are arithmetic mean roughness (Ra) and average surface roughness (Sa). Atomic force microscopy allows 3D imaging of surfaces at nanometer resolution, interprets the image in the form of quantitative data, and therefore serves as helpful and reliable in assessing the surface roughness of different materials [14-16]. Since several polishing systems are available for composites and very limited information is known about pre-contoured cervical matrix and its impact on surface roughness, this study aims to evaluate the surface roughness exhibited by pre-contoured cervical matrix systems and to compare it with abrasive disk polishing systems, which are most commonly used in recent times. The primary objective of the study was to compare the surface roughness exhibited by class V composite restorations (using microhybrid and nanohybrid types of composites) restored using pre-contoured cervical matrix systems and polished using abrasive disk polishing.

## Materials and methods

The sample size was determined using G*Power software version 3.1.9.6 (Heinrich Heine University Düsseldorf, Düsseldorf, Germany). The sample size was estimated based on the previous study [7]. A total sample of 20 (1-β = 90%, α = 0.05, d= 1.6) was obtained based on the calculation.

Twenty extracted, clean, non-carious, human maxillary anterior teeth were collected from the Department of Oral Surgery at the Saveetha Dental College and Hospitals, Chennai, India. One operator performed all the procedures to reduce operator bias. The study investigated the surface roughness of class V cavities restored with a pre-contoured cervical matrix system (BlueView Cervical Matrices, Garrison Dental Solutions, Spring Lake, USA) (n=10) compared to cavities restored and polished using a super-snap polishing system (Shofu Super-Snap Rotary Polishing Instruments-Mini Kit, Shofu Dental India, New Delhi, India) (n=10). Two different composites (n=5 per group) were used: microhybrid (Ivoclar Te-Econom Plus Composite, Ivoclar Vivadent, Schaan, Liechtenstein) and nanohybrid (Dentsply ceram.x, Dentsply, Konstanz, Germany) composites of commercially available composite brands (Table 1).

**Table 1:** Composition of the composites used in the study TEGDMA: triethylene glycol dimethacrylate; Bis-EMA: bisphenol A diglycidyl methacrylate ethoxylated

Composite	Te-Econom plus	Ceram.x
Manufacturer	Ivoclar Vivadent, Schaan, Liechtenstein (L6403)	Dentsply, Konstanz, Germany
Resin matrix	Dimethacrylate, TEGDMA	Polyurethane methacrylate, Bis-EMA and TEGDMA, Methacrylic polysiloxane nanoparticles
Filler composition	Barium glass, ytterbium trifluoride, silicon dioxide, and mixed oxide (0.04 -7 µm)	Spherical, pre-polymerized Sphere TEC™M fillers (d3,50=15 µm), non-agglomerated barium glass, and ytterbium fluoride
Type of filler	Microhybrid	Nanohybrid
Filler content by volume/weight %	60/76	61/79

One group of samples was restored with the help of pre-contoured cervical matrices and received no finishing treatment after being cured under the cervical matrix. The other group was restored using conventional techniques without the use of a matrix and polished using super-snap polishing discs.

Standardized class V cavity preparation was done, followed by etching with 37% orthophosphoric acid and rinsing. A universal bonding agent was applied and light-cured, and the cavities were filled and condensed well with microhybrid (n=5 per group) and nanohybrid (n=5 per group) composites of two different brands (Ivoclar and Dentsply). Ten samples were restored with the pre-contoured cervical matrix system, and 10 samples were polished using the multi-step super-snap system. These were then embedded in cold-cured acrylic resin blocks, and the images were assessed under an atomic force microscope (Step 700 Noise Control, Anton Paar, Graz, Austria). Nanosurf Nanite Atomic Force Microscope (AFM) software, Nanosurf Control C-3000 (Nanosurf AG, Liestal, Switzerland), was used in contact mode. It consists of a tip that contacts the surface of the sample and provides the Ra and Sa values at the nanoscale. The resolution of the image produced was 25x25 μm, with 128 lines and dots scanned in 0.78 seconds per line. The surface roughness was measured for four groups of samples, consisting of five samples in each group; Group A: microhybrid composite polished with a super-snap polishing system; Group B: microhybrid composite restored with pre-contoured cervical matrix; Group C: nanohybrid composite polished with a super-snap polishing system; and Group D: nanohybrid composite restored with a pre-contoured cervical matrix.

The data were tabulated in a Microsoft Excel sheet (Microsoft Corp., Redmond, WA, USA), and data analysis was performed using IBM SPSS Statistics software for Windows version 23.0 (IBM Corp., Armonk, NY, USA). The Shapiro-Wilk test was employed to check the normality of the data. The one-way analysis of variance (ANOVA) test and Tukey's Honest Significant Difference test (Tukey's HSD) were performed to obtain the results of the study.

## Results

The Ra and Sa values of each of the samples obtained through AFM were compared. The Ra values indicate the arithmetic average of the absolute values of the roughness profile ordinates of 128 lines present in the sample being studied, and the Sa values indicate the average surface area roughness. The Ra values of the samples tested were found to be Group A (332.58 nm), Group B (183.09 nm), Group C (290.55 nm), and Group D (57.84 nm). Meanwhile, the Sa values of the samples tested were found to be Group A (313.97 nm), Group B (159.41 nm), Group C (337.47 nm), and Group D (33.87 nm) (Table 2).

**Table 2:** Mean distribution of Ra and Sa among the groups derived by one-way ANOVA Ra: average line roughness; Sa: average surface roughness

Variable	Mean ± SD				p-value
	Group A	Group B	Group C	Group D	
Ra	332.6 ± 0.445	183.1 ± 0.200	290.6 ± 0.544	57.84 ± 0.086	0.000
Sa	313.9 ± 0.910	159.4 ± 0.505	337.5 ± 0.403	33.87 ± 0.171	0.000

**Figure 1:**
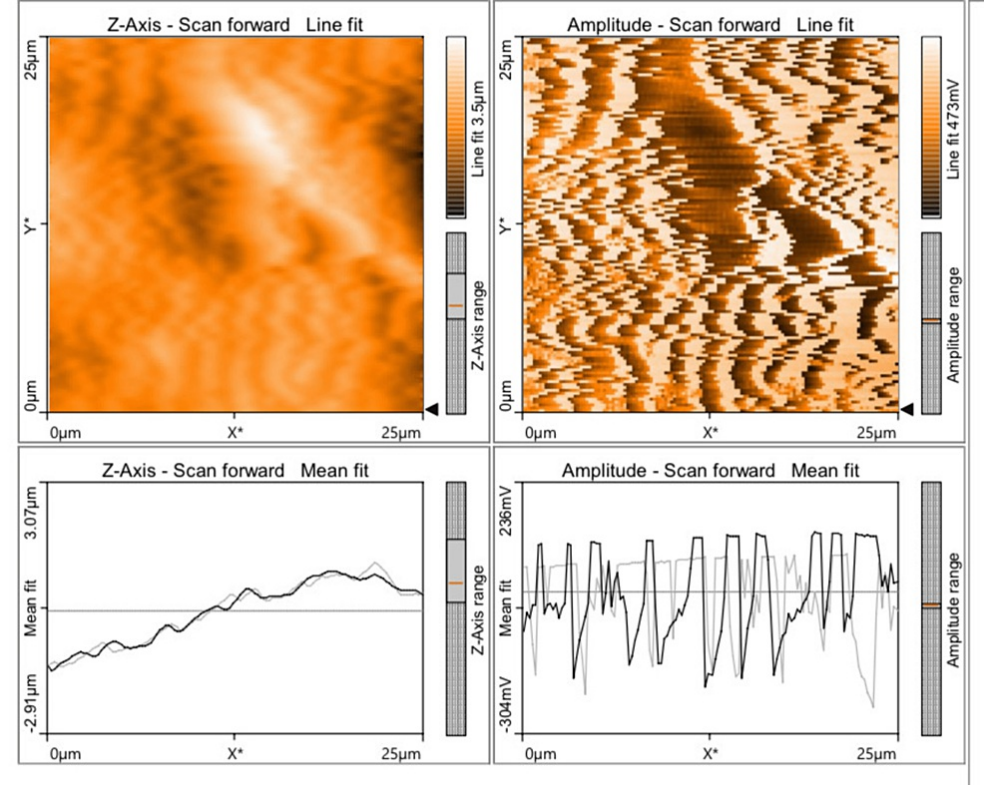
AFM view of a nanohybrid composite restored with a pre-contoured cervical matrix system AFM: atomic force microscope

**Figure 2:**
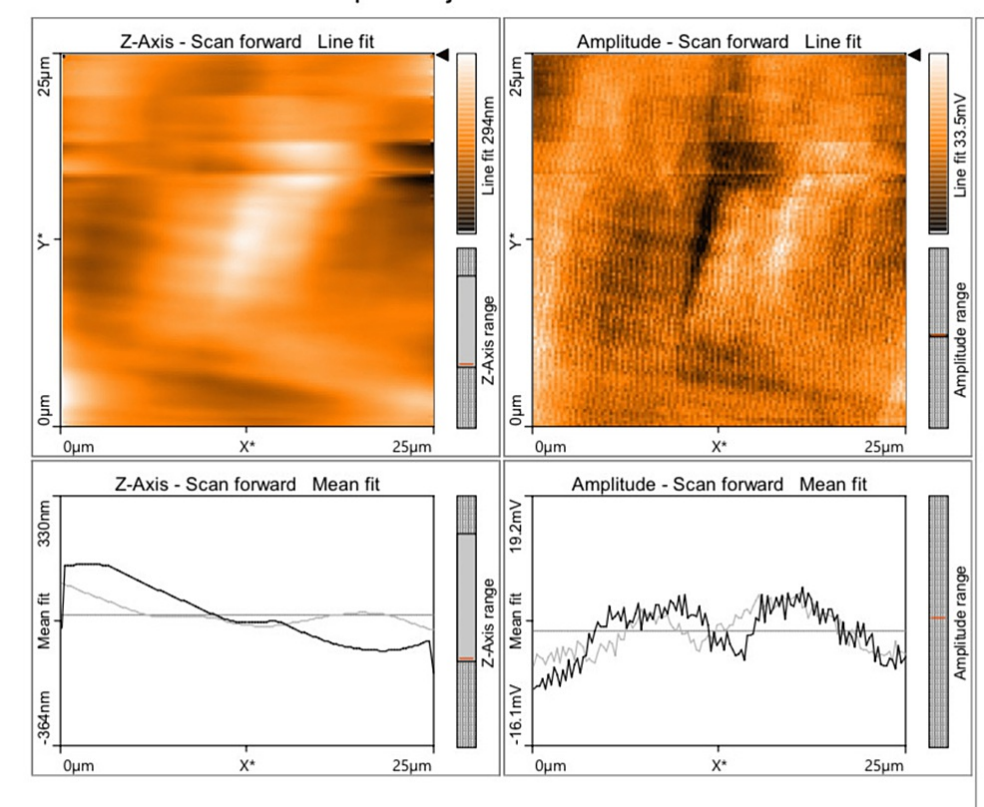
AFM view of a microhybrid composite restored using a pre-contoured cervical matrix system AFM: atomic force microscope

**Figure 3:**
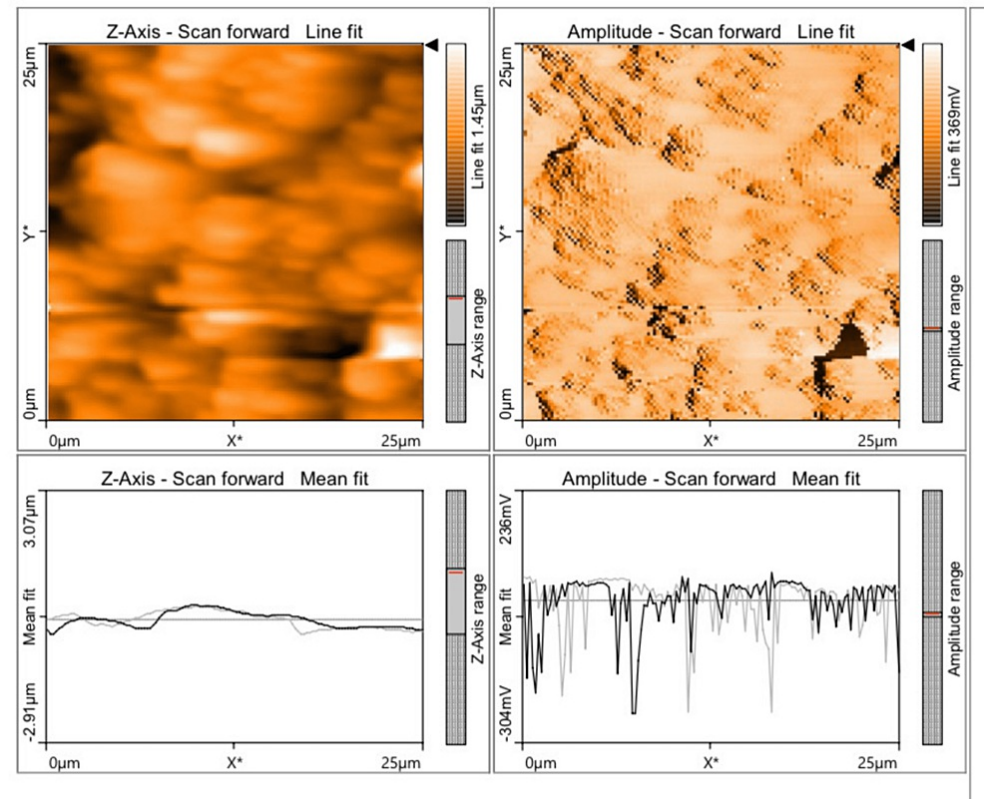
AFM view of a nanohybid composite polished using the super-snap polishing system AFM: atomic force microscope

**Figure 4:**
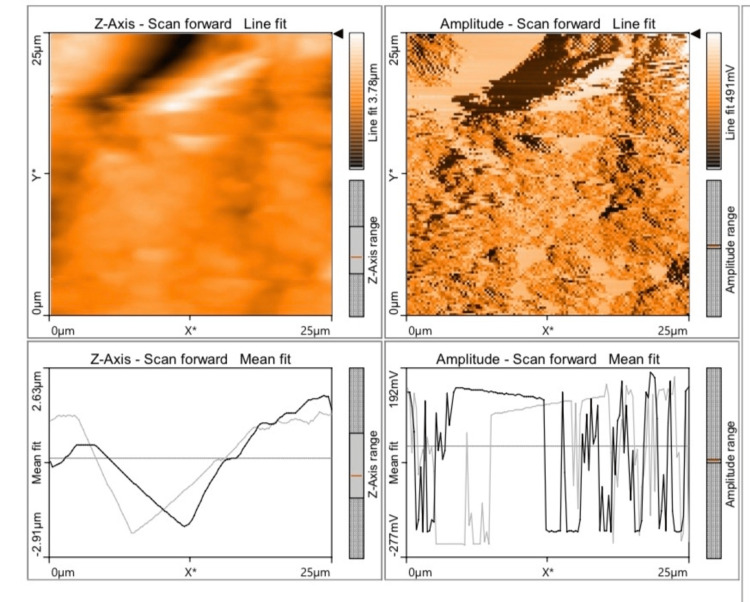
AFM view of a microhybrid composite polished using the super-snap polishing system AFM: atomic force microscope

The mean value of discrepancy for Ra and Sa values was found to be the least for Group D (57.84 and 33.87) (Figure 1), followed by Group B (183.1 and 159.4) (Figure 2), Group C (290.6 and 337.5) (Figure 3), and Group A (332.6 and 313.9) (Figure 4), which indicates that Group D (a nanohybrid composite restored with a pre-contoured matrix) exhibited the least Ra and Sa surface roughness values.

The highest surface roughness was exhibited by Group A (a microhybrid composite polished with a super-snap polishing system). The p-value for all the groups in relation to Ra and Sa values was found to be 0.000 (p<0.05), which is statistically significant. A Tukey HSD post hoc test was done for pairwise comparison of surface roughness parameters (Ra and Sa) among the four groups studied. The p-value for all the comparisons was 0.000 (p<0.05), which is statistically significant. The Ra and Sa values of Groups A and B were compared, and their mean differences were found to be 149.5 and 154.6, respectively, with a p-value of 0.000 (p<0.05), which is statistically significant. The Ra and Sa values of Groups A and C were compared, and their mean differences were found to be 42.03 and -23.51, respectively, with a p-value of 0.000 (p<0.05), which is statistically significant. The negative value indicates that Group C had lower roughness values than Group A.

The Ra and Sa values of Groups A and D were compared, and their mean differences were found to be +274.7 and +280.1, respectively, with a p-value of 0.000 (p<0.05), which was statistically significant. The Ra and Sa values of Groups B and C were compared, and their mean differences were found to be -107.5 and -178.1, respectively, with a p-value of 0.000 (p<0.05), which was statistically significant. The negative value indicates that Group C had lower roughness values than Group B. The Ra and Sa values of Groups B and D were compared, and their mean difference was found to be +125.3 and +125.5, respectively, with a p-value of 0.000 (p<0.05), which was statistically significant. The Ra and Sa values of Groups C and D were compared, and their mean differences were found to be +232.7 and +303.6, respectively, with a p-value of 0.000 (p<0.05), which was statistically significant (Table 3).

**Table 3:** Pair-wise comparison of Ra and Sa among the groups Ra: average line roughness; Sa: average surface roughness

Variable	Pairs	Mean difference	p-value
Ra	Group A vs. Group B	149.5	0.000
	Group A vs. Group C	42.03	0.000
	Group A vs. Group D	274.7	0.000
	Group B vs. Group C	-107.5	0.000
	Group B vs. Group D	125.3	0.000
	Group C vs. Group D	232.7	0.000
Sa	Group A vs. Group B	154.6	0.000
	Group A vs. Group C	-23.51	0.000
	Group A vs. Group D	280.1	0.000
	Group B vs. Group C	-178.1	0.000
	Group B vs. Group D	125.5	0.000
	Group C vs. Group D	303.6	0.000

The Ra and Sa values were the lowest for Group D, followed by Group B, Group C, and Group A. Both values were lower for groups that employed the pre-contoured cervical matrix than for groups that employed the super-snap polishing system.

## Discussion

In the present study, among the tested groups, regardless of the type of composite used, both the values (Ra and Sa) were lower for the cavities restored using the pre-contoured cervical matrix system when compared to those of the super-snap polishing system. Microhybrid composites are widely used for their mechanical, physical, and polishing properties. As polishing could be regarded as a grinding process, fillers with greater size may lose the retention of the resin matrix and subsequently fall off, leaving more pits and dents on the surfaces. Therefore, optimal surface smoothness could be hardly achieved. However, with the introduction of nanofillers, which have particle sizes of 1-100 nm, this issue could be addressed as they combine the advantages of hybrid and microfilled composites as they exhibit low shrinkage, good mechanical properties, better polish and gloss, increased retention, and wear resistance, which is at least equivalent to or may surpass those of the existing materials [17].

Concerning the composite used, nanohybrid composites exhibited lesser Ra and Sa values than microhybrid composites. The resin composite restoration, which is well adapted and contoured with a pre-contoured matrix and remains untouched by cutting instruments, abrasive discs or cups, and finishing instruments, will have filler particles that are not abraded away from the resin matrix, resulting in a smoother surface. Hence, the use of pre-contoured cervical matrices is advantageous in this aspect, wherein no abrasive discs or burs are employed for polishing the restoration, and it consistently helps to form an adequate seal in the critical gingival areas being restored, eliminating the need for difficult operator shaping and finishing [18]. It has also been reported that upon polishing, larger filler particles protrude from the resin matrix, thereby increasing the Ra value [19,20].

In a study conducted by Hassan AM and colleagues, they assessed three different resin composites (heliomolar flow, TPH spectrum, and Tetric Ceram HB) alongside three distinct polishing systems (Astropol, Enhance, and Soflex) and found that the control group that received no polishing and was just cured with mylar strip exhibited the lowest Ra values in the profilometer than the other groups, which had no statistically significant difference. The samples used in this study were flat, which cannot exist clinically [21]. Yap AU et al. compared the surface roughness of two different restorative materials polished with single-step Matrix strips, two-step rubber discs, and graded abrasive discs (super-snap system) using a profilometer and found that for both the restorative materials used, the smoothest surface was obtained with matrix strips [19]. Müllejans R. et al., in their study, reported that the class II restorations that were done using transparent matrices had higher overhang formation when compared with metal matrices, especially in the case of flowable composites [22].

In a study conducted by Jung M et al., the authors compared the surface roughness of four nanocomposites (Premise (KerrHawe), Tetric EvoCeram (Ivoclar Vivadent), Filtek Supreme (3M ESPE), and Ceram X Duo (Dentsply)) and one hybrid composite (Herculite XRV (KerrHawe)) after polishing with three different polishing systems (flexible Sof-Lex discs (3M ESPE), the Astropol system (Ivoclar Vivadent), OptiShine brushes (KerrHawe), and the Enhance/PoGo system (Dentsply). They found that two of the four nanocomposites were significantly smoother than the hybrid composite, while the remaining two had a surface quality similar to that of a hybrid composite [23].

In our study, we have employed the use of packable composites that are well condensed into the cavity to avoid underfilled margins, and the use of pre-contoured matrices helps in superior adaptability to the natural contour of the tooth structure, which enables us to avoid excess material buildup. The current study overcomes this limitation by having the sample (composite resin) present in the tooth itself to follow the tooth morphology. The current study has some limitations. This is an in vitro study conducted in a controlled laboratory environment, which may not mimic the complex and dynamic oral environment. This study compares only two types of composites and polishing methods. More extensive study designs, comparing various available materials, and polishing systems are necessary to facilitate the practical application of this method. Additional in vivo investigations will be essential to validate the findings.

## Conclusions

Based on the procedure performed and the results obtained, it can be concluded that resin composites restored using single-step, pre-contoured cervical matrices exhibited a lower degree of surface roughness than that of serial abrasive disk polishing. Among the types of composite resins tested, nanohybrid composites showed lesser surface roughness than microhybrid composites. Moreover, atomic force microscopy helped in better visualization and distinguishability of surface roughness across the tested groups.
